# Effect of Humidity on CO_2_/N_2_ and CO_2_/CH_4_ Separation Using Novel Robust Mixed Matrix Composite Hollow Fiber Membranes: Experimental and Model Evaluation

**DOI:** 10.3390/membranes10010006

**Published:** 2019-12-30

**Authors:** Clara Casado-Coterillo, Ana Fernández-Barquín, Angel Irabien

**Affiliations:** Department of Chemical and Biomolecular Engineering, Universidad de Cantabria, s/n, 39005 Santander, Spain; fbarquina@unican.es (A.F.-B.); irabienj@unican.es (A.I.)

**Keywords:** mixed matrix, composite hollow fiber membrane, CO_2_ separation, humid gas streams, modeling validation

## Abstract

In this work, the performance of new robust mixed matrix composite hollow fiber (MMCHF) membranes with a different selective layer composition is evaluated in the absence and presence of water vapor in CO_2_/N_2_ and CO_2_/CH_4_ separation. The selective layer of these membranes is made of highly permeable hydrophobic poly(trimethyl-1-silylpropine) (PTMSP) and hydrophilic chitosan-ionic liquid (IL-CS) hybrid matrices, respectively, filled with hydrophilic zeolite 4A particles in the first case and HKUST-1 nanoparticles in the second, coated over compatible supports. The effect of water vapor in the feed or using a commercial hydrophobic PDMSXA-10 HF membrane has also been studied for comparison. Mixed gas separation experiments were performed at values of 0 and 50% relative humidity (RH) in the feed and varying CO_2_ concentration in N_2_ and CH_4_, respectively. The performance has been validated by a simple mathematical model considering the effect of temperature and relative humidity on membrane permeability.

## 1. Introduction

Membrane technology for CO_2_ separation from other gases, especially N_2_ and CH_4_, faces challenges to upgrade to large scale, partly due to the uncertainty of the behavior in the presence of impurities such as water vapor in real gas separation [1,2,3,4,5,6], and partly to the trade-off between permeability and selectivity in a gas pair separation that has been often proposed to be overcome by emerging materials [7]. Among these, mixed matrix membranes (MMMs) combine the processability of polymers with the molecular sieving effect of inorganic fillers, and have been investigated intensively for light gas separation [8]. Despite the achievements carried out in material development in the last decades at lab scale, there is still a gap between lab and practical conditions because of the difficulty of fabrication of membranes from new materials [9]. Multilayer composite membranes offer the possibility to optimize membrane layer materials independently and reduce the overall transport resistance by coating ultrathin highly selective and permeable layers on mechanically robust and processable supports [10].

This way, a multilayer composite hollow fiber (CHF) approach allows the transfer of the selective layer properties to other geometries [11], which could be more easily implemented at a large scale, by dip-coating the selective material as a thin layer on a robust support, which is simpler than wet-dry phase inversion spinning [12,13], co-extruding a thin ion-exchange hydrophilic polymer high performance material on hydrophobic polysulfone (PSf) [14], or growing zeolites in a polymer support [15]. One of the most important impurities in CO_2_ separation is water vapor because the relative humidity of the feed gas [16]. It has been suggested that water vapor can affect the membrane performance differently depending on the hydrophobic or hydrophilic character of the selective layer material and the affinity of H_2_O with the gas penetrants [17,18]. If the membrane is hydrophobic, water has been reported to reduce the solubility of gases through competitive effects, lower the free volume of the polymer or reduce diffusivity by blocking effects of the pores of hydrophilic pores in MMMs [19]. If the membrane were hydrophilic, the interaction with water can be strong and increasing when exposed to humid conditions, the gas permeance being due to the increased gas diffusivity [20]. This can be prevented by coating a layer of a different character on top of the composite membrane, the support, or by combining hydrophilic and hydrophobic components in the selective layer [21].

Defects that make the membrane unselective also need to be controlled [22], but intrusion of the coated layer in the pores of the support has to be avoided to maintain the high flux needed in CO_2_ separation. This has been attempted by pre-wetting the substrate in water [17], adding a hydrophobic gutter layer of polydimethylsiloxane (PDMS) [23], or poly(trimethyl silyl-1-propyne) (PTMSP) between a hybrid hydrophilic selective layer and the porous support [24,25], or coating a protective layer of a high free volume AF2400 [22] or hydrophilic chitosan (CS) biopolymer [26,27] on porous hydrophilic substrates to simultaneously enhance both permeance and selectivity in CO_2_ separation.

In this work, we study the experimental separation of CO_2_/N_2_ and CO_2_/CH_4_ mixtures in dry and wet conditions, as a function of feed concentration and the composition of the selective layer of hydrophobic PTMSP/P84 and hydrophilic IL-CS/PSf composite hollow fiber (CHF) membranes, both highly CO_2_ permeable and thermally robust polymers. The selective layer has also been modified by the MMM concept, using compatible fillers that enhanced the permselectivity and mechanical resistance of the pure polymer materials [28,29], such as HKUST-1-IL-CS and Zeolite 4A-PTMSP MMMs, even when transferred to hollow fiber geometry at increasing operating temperatures by an appropriate choice of compatible selective layer components and supports [30], to create mixed matrix composite hollow fiber (MMCHF) membranes. The separation performance of these membranes was evaluated in this work by the adaptation of a simple mathematical model developed previously for the CO_2_/N_2_ separation as a function of temperature, concentration, and number of membrane modules in series [31].

## 2. Materials and Methods

The preparation and morphological characterization of the CHF and MMCHF membranes studied here was presented in a previous work [30]. PTMSP and 20 wt.% zeolite A-PTMSP MMCHF membranes were prepared by coating the selective solution on the outer side of a P84 HF support, with the ends covered to prevent penetration in the lumen side. Likewise, IL-CS and 5 wt.% HKUST-1 MMCHF membranes were prepared on the outer side of a PSf HF support. In order to do these, PTMSP was purchased from ABCR (Karlsruhe, Germany), CS, IL and Zeolite 4A from Aldrich (Madrid, Spain), while HKUST-1 nanoparticles were supplied by the University of Zaragoza [29] and the P84 and PSf HF supports by Tecnalia [30].

**Figure 1:**
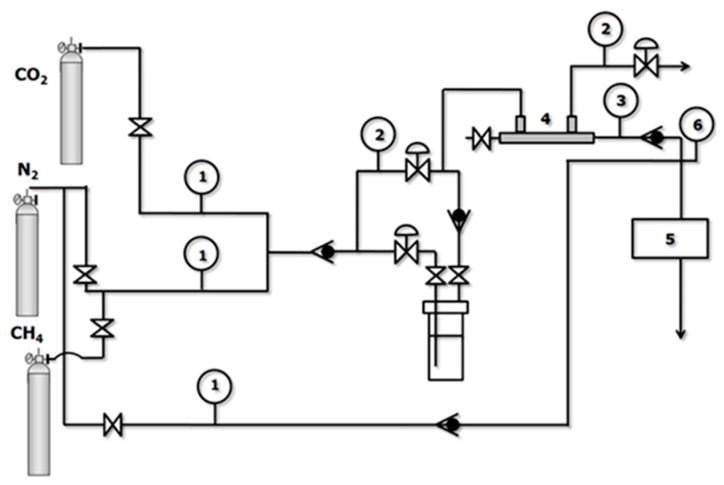
Experimental setup. (1) Mass flow meters, (2) pressure regulators, (3) pressure gauges, (4) HF membrane module, (5) analyzer, (6) bubble flowmeter.

**Figure 2:**
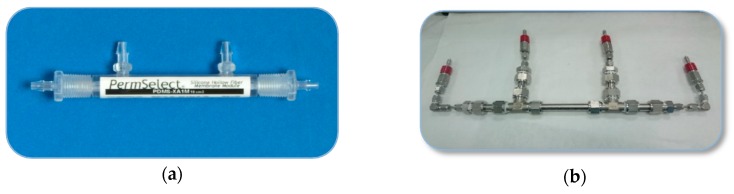
Photographs of the commercial PDMSXA-10 HF membrane module and the home-made stainless-steel modules used to measure the separation performance of the CHF and MMCHF membranes in our laboratory.

The performance of the membranes for the separation of CO_2_/N_2_ and CO_2_/CH_4_ mixtures was experimentally evaluated in a home-made separation setup at 0% and 50% relative humidity (RH) (Figure 1). To perform the wet gas experiments, the feed gas at the operating pressure and temperature was half-passed through a water tank, as shown in the Figure 1, before being introduced to the shell side of the hollow fiber membranes. The stop valves in Figure 1 prevented the entrance of liquid water to the membrane module, allowing comparison of the behavior of the membrane in the presence and absence of water vapor [32]. A commercial PDMSXA-10 HF membrane (Permsilicone^®^) has also been tested for comparison purposes. The membrane modules can be seen in the photographs in Figure 2, where Figure 2a is the commercial module and Figure 2b the module where the lab-made CHF and MMCHF membranes were placed for testing. The experiments were performed at room temperature (293K) and 4.5 bar absolute feed pressure, commonly encountered conditions in the characterization of MMMs and thin-film composite (TFC) membranes for CO_2_ separation reported in literature [20,33,34,35]. The sequence of experiments conducted in the separation plant is presented in Table 1.

Once the membrane performance reached a steady state, the permeate was measured using a bubble flow meter at the end of the system (6) at least 3 times over for about 1 h to confirm the membrane stability at a given operating condition. Stable performance was attained after 3 h in dry conditions. The composition of the permeate was determined by a gas analyzer (BIOGAS5000, Geotech, USA, purchased from Fonotest S.L., Madrid, Spain).

The permeance of gas *i*, was calculated as

(1) $$\left(\frac{P_{i}}{t}\right)=\frac{Q\times y_{i}}{A\Delta p_{i}}$$

Permeance is expressed as usual in terms of GPU (1 GPU = 10^−6^ cm^3^ (STP) cm^−2^ s^−1^ cmHg^−1^), where *i* is referred to the permeating gas molecule, Δ*p_i_* the partial pressure difference for the gas component *i* across the membrane, *A* the effective area of the membrane, *t* the effective layer thickness for the separation and *Q* the permeate flow rate (cm^3^/s) at measurement pressure and temperature conditions. The effective area of the MMCHF membranes was 2.2 cm^2^. The effective area of the commercial PDMSXA-10 HF membrane was 10 cm^2^, the smallest we have found to compare with the lab-made MMCHF membranes.

The selectivity was calculated as the ratio between the permeance of the fast gas *i*, i.e., CO_2_, and the slow gas, *j*, in this work, N_2_ or CH_4_, respectively, as expressed by

(2) $$\beta_{ij}=\frac{{\left(P/t\right)}_{i}}{{\left(P/t\right)}_{j}}$$

where *P* is the intrinsic selective material permeability measured by single gas permeation through the self-standing materials in previous works [28,29], *t* the effective layer thickness, taken from the previous work as 0.5 ± 0.1, 1.9 ± 0.5, 7.2 ± 1.7 and 6.9 ± 1.5 µm, for IL-CS/PSf, 5wt.% HKUST-1-IL-CS/PSf, PTMSP/P84 and 20 wt.% zeolite A-PTMSP/P84 membranes, respectively. These data are reported in a previous work [30].

The influence of membrane composition, type of separation and feed concentration on the separation performance have been evaluated experimentally and validated a simple mathematical model developed in a previous work using Aspen Custom Modeler^®^ [31]. On the one hand, this model has been updated in this work to take into account the change of geometry by updating the model assumptions as in [36] for thermally resistant hollow fiber membranes:

- ideal gas behavior,
- no deformation of the hollow fiber or gas leakage losses,
- the inner and outer diameters and of the hollow fibers thickness of the selective layer are uniform for the whole effective length of the module,
- the effect of concentration polarization is negligible,
- the permeance depends on the feed conditions, and can be estimated based on correlations dependent on conditions including pressure, flowrate, and composition, and
- the pressure drop is negligible on both the permeate and feed sides [37].

Furthermore, the effect of the selective layer and support is taken into account using the resistance-in-series approach [38,39,40], as

(3) $${\left(\frac{t}{P}\right)}_{global}={\left(\frac{t}{P}\right)}_{support}+{\left(\frac{t}{P}\right)}_{selective\;layer}$$

On the other hand, the model has been also updated to account for the effect of humidity by using the NELF-based solubility-diffusivity approach with two adjustable parameters A and B depending on the selective membrane material properties for each penetrating gas molecule, as [21,41].

(4) P(a)=D(a)·S(a)=A·exp(−BFFV(a))·S(a)

where *FFV*(*a*) is the fraction of free volume available for gas transport as a function of the water activity, *a*, in the system, which accounted for the presence of water vapor in the feed. The values of A and B for PTMSP and Zeolite A-PTMSP MMM were reported elsewhere [21], and those used for IL-CS and HKUST-1/IL-CS MMM were estimated from the permeability values measured in the laboratory in wet and dry conditions.

## 3. Results and Discussion

### 3.1. Experimental Evaluation

The experimental results of the CO_2_/N_2_ and CO_2_/CH_4_ gas mixture separations in dry and wet conditions for the different membranes are discussed in the following lines.

**Figure 3:**
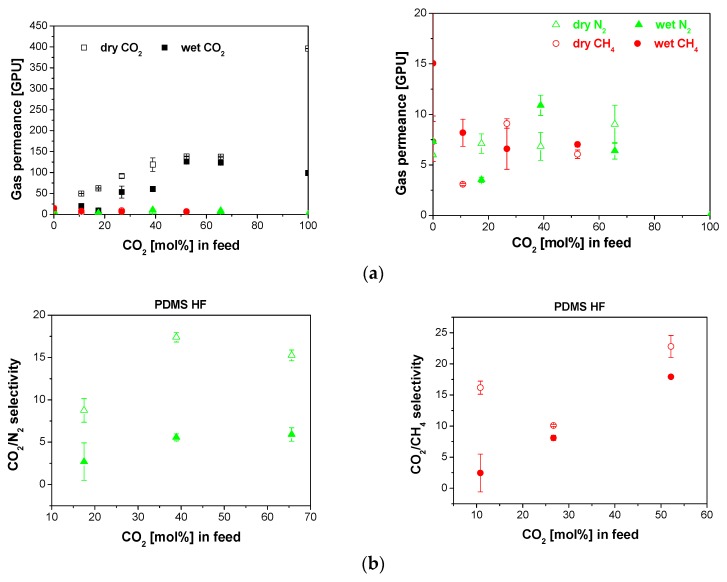
Gas permeance (**a**) and selectivity (**b**) obtained for the separation of CO_2_/N_2_ (left) and CO_2_/CH_4_ (right) mixtures through the commercial PDMSXA-10 HF membrane in dry (void symbols) and wet (full symbols). The right side of figure (**a**) shows the trend in the slow gas (N_2_, CH_4_) permeance.

The separation performance through the commercial PDMS10XA-10 HF membrane is shown in Figure 3. The CO_2_ permeance through this rubbery hydrophobic membrane increased with increasing CO_2_ content in the feed mixture, while the N_2_ and CH_4_ permeances did not decrease. The reduction of the low permeating gas (N_2_, CH_4_) permeance was less significant than that of CO_2_, due to differences in condensability (boiling point of N_2_ 77 K, CH_4_, 112 K and CO_2_, 126 K) and kinetic diameter (0.38 nm, 0.34 and 0.33 nm, for CH_4_, N_2_ and CO_2_, respectively). Thus, the differences of CO_2_/CH_4_ and CO_2_/N_2_ selectivities in Figure 3b, may be attributed to competing and synergistic interactions between the penetrants and the polymer selective layer. The presence of water vapor, reduced the CO_2_/N_2_ selectivity more than the CO_2_/CH_4_ selectivity, as observed in Figure 3b, because the slow gas permeance decreased at increasing CO_2_ concentration in the feed, due to the favorable competition for adsorption coverage sites in the membrane matrix. Therefore, the selectivity generally increased in the presence of water vapor, i.e., up to around 40% in the case of CO_2_/N_2_ selectivity, in agreement with the partial recovery of the slow gas permeance recovered after a series of experiments in the presence of water vapor, as observed by Chenar et al. [18] for commercial polymer HF membranes.

**Figure 4:**
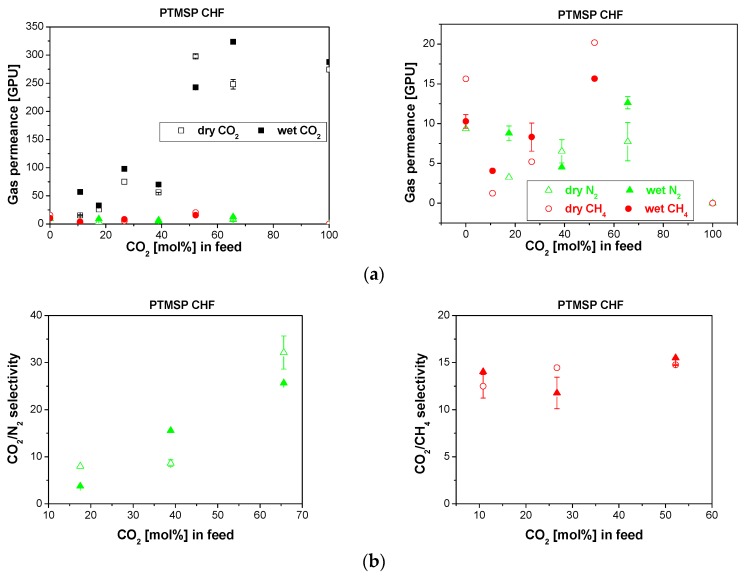
Gas permeance (**a**) and selectivity (**b**) obtained for the separation of CO_2_/N_2_ (left) and CO_2_/CH_4_ (right) mixtures through the PTMSP/P84 CHF membrane in dry (void symbols) and wet (full symbols). The right side of figure (**a**) shows the trend in the slow gas (N_2_, CH_4_) permeance.

In Figure 4 the CO_2_/N_2_ and CO_2_/CH_4_ mixtures separation performance in the absence and presence of water vapor the PTMSP/P84 CHF membranes prepared in the laboratory, is presented. Similar to the commercial PDMSXA-10 HF membrane in Figure 3, the presence of water vapor in the gas stream hardly decreases the CO_2_ permeance or the selectivity of the PTMSP/P84 CHF membrane in Figure 4. This was again attributed to the combined effect between competitive sorption and transport, perhaps due to the high free volume and rigid structure of PTMSP [18,19]. The selectivity values in Figure 4b were of the same order of magnitude as other PTMSP/P84 CHF membranes reported in literature [19]. Since the gas permeance and selectivity were not altered by the presence of water vapor, it was expected that the integrity of the polymer coating was preserved through the experiments [42]. This observation agreed with other authors’ results on membranes coated with PDMS to prevent the water molecules being trapped in the hydrophilic support [19]. When those membranes showed the same performance wet as dry [30], this was considered as an indication of the defect ratio of the CHF membrane [22].

**Figure 5:**
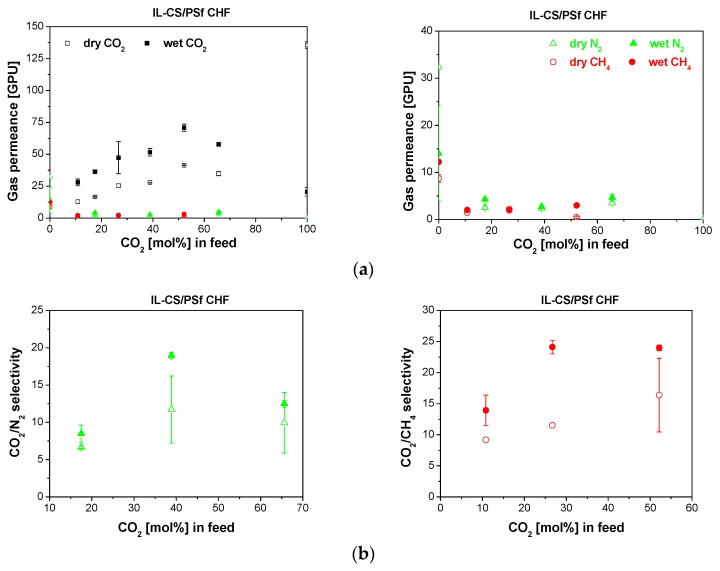
Gas permeance (**a**) and selectivity (**b**) obtained for the separation of CO_2_/N_2_ (left) and CO_2_/CH_4_ (right) mixtures through the IL-CS/PSf CHF membrane in dry (void symbols) and wet (full symbols). The right side of figure (**a**) shows the trend in the slow gas (N_2_, CH_4_) permeance.

The behavioral trend observed in Figure 3 and Figure 4 was opposed to that of the IL-CS/PSf CHF membrane in Figure 5, where the presence of water vapor in the feed increased the CO_2_ permeance while diminishing the fluxes of CH_4_ and N_2_, probably because of swelling of the IL-CS matrix, thus revealing the hydrophilic nature of the selective IL-CS layer [29].

Although the selectivity through the hydrophilic IL-CS/PSf membrane increased at low CO_2_ concentration in the feed by the effect of water vapor, for CO_2_/N_2_ and CO_2_/CH_4_ separation, the selectivity declined at higher concentration probably because of the carrier effect of CO_2_ and water through the membrane [43]. Highly hydrophilic membranes have been observed to interact strongly with water vapor, causing a drop in the performance in wet compared to dry conditions [35]. This is even more remarked at increasing CO_2_ feed concentration because of the interaction of CO_2_ with water molecules [33].

In fact, the hydrophilic IL-CS/PSf CHF shows a slight increase of CO_2_ selectivity and CO_2_ permeance in the presence of water vapor conditions, compared to dry, at 25 wt.% CO_2_ feed concentration, which may be attributed to the role of the [emim][Ac] ionic liquid in the selective layer upon wet gas permeation experiments, as reported elsewhere [35]. Fam et al. controlled the swelling of Pebax-IL CHF membranes by adding graphene oxide (GO) sheets with high water affinity [20]. In our laboratory, we attempted doing so by adding nanoparticles with high water and CO_2_ affinity [44,45]. Thus, as previously reported [29], the IL is also acting as a binder or void filler [46] with the HKUST-1 nanoparticles in the selective layer of 5 wt.% HKUST-1-IL-CS/PSf MMCHFs, leading to higher increases in CO_2_ permeance and selectivity than those observed for the rest of the MMCHF membranes in this work. This agrees with the observations reported for thermally arranged HF membranes on the separation of simulated flue gas mixtures [47], and it may be attributed to the combined hydrophilic and hydrophobic nature of the selective membrane layer and the successful coating of the effective length of the support, providing a lower defect ratio [17].

**Figure 6:**
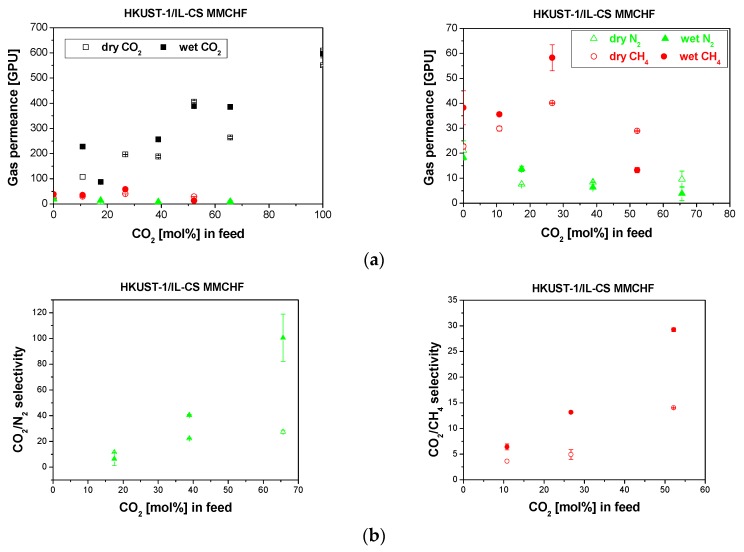
Gas permeance (**a**) and selectivity (**b**) obtained for the separation of CO_2_/N_2_ (left) and CO_2_/CH_4_ (right) mixtures through the HKUST-1/IL-CS/PSf MMCHF membrane in dry (void symbols) and wet (full symbols). The right side of figure (**a**) shows the trend in the slow gas (N_2_, CH_4_) permeance.

The experimental results of the CO_2_ separation performance through our HKUST-1-IL.CS/PSf MMCHF membranes are plotted in Figure 6. Interestingly, the CO_2_ permeance through the HKUST-1/IL-CS MMCHF membrane in Figure 6a increased dramatically compared to the IL-CS/PSf HF membrane in Figure 5a, and although the CO_2_ permeance decreased slightly in wet CO_2_/N_2_ separation, compared to dry conditions, the values of these membranes were the highest obtained for all the membranes reported in this work.

The selectivity of the membranes is increased from 10–20 for the polymer IL-CS/PSf CHF in Figure 5, to 40–50 for the MMCHF in Figure 6. This values are in agreement with other MMCHF membranes reported in literature, as observed by other authors for hydrophilic amine-modified SAPO-34-IL/Pebax-PEGDME MMCHF [46], or GO-IL/PTMSP/PVDF MMCHF [20], in 20:80 (%) CO_2_:N_2_ and CO_2_:CH_4_ mixture separation, and thereby attributed to the competitive sorption between CH_4_ and CO_2_.

**Figure 7:**
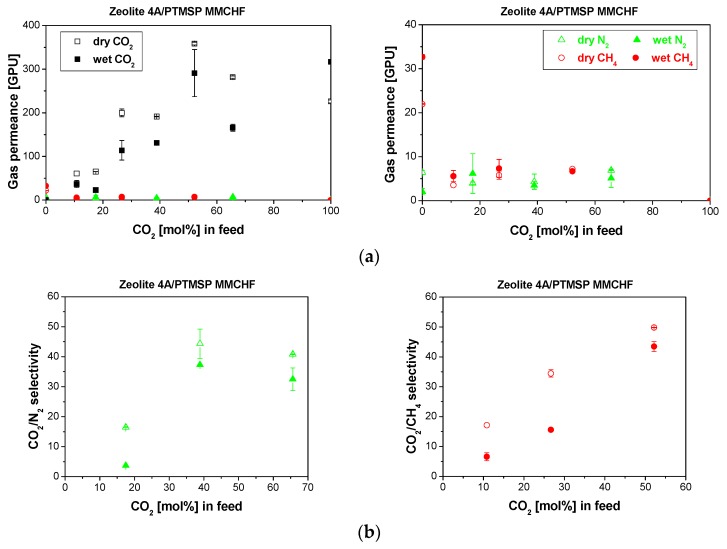
Gas permeance (**a**) and selectivity (**b**) obtained for the separation of CO_2_/N_2_ (left) and CO_2_/CH_4_ (right) mixtures through the Zeolite A/PTMSP/P84 MMCHF membrane in dry (void symbols) and wet (full symbols). The right side of figure (**a**) shows the trend in the slow gas (N_2_, CH_4_) permeance.

Hydrophilic zeolite fillers and hydrophilic glassy polymers usually have to be surface-modified to increase their compatibility. The combination of hydrophilic zeolite fillers and hydrophobic polymer matrices in the selective layer influenced the CO_2_ permeance and selectivity of Zeolite A/PTMSP MMMs in the presence of water vapor [21], and is appreciated for the Zeolite A-PTMSP/P84 MMCHF in Figure 7. This was attributed to the effect of the introduction of the zeolite A particles in the PTMSP matrix [21], by simultaneously influencing the plasticization of the PTMSP polymer and the water sorption and molecular sieving of the zeolite A. The water uptake was increased from 9.5 ± 5% for PTMSP to 33 ± 6% for the 20 wt.% Zeolite A-PTMSP MMM. This was the cause of the decrease in CO_2_ permeance in wet conditions. The N_2_ and CH_4_ permeance were less influenced by water vapor presence than they were for the hydrophobic PTMSP/P84 CHF and PDMSXA-10 HF membranes, and so the CO_2_/N_2_ and CO_2_/CH_4_ selectivity increased around 34% and 46%, respectively, from dry to humid conditions.

In the feed. The combination of hydrophilic Zeolite A and PTMSP in the selective layer of the composite membrane provided a stable performance at 25 wt.% CO_2_ concentration in the feed. Especially, the constant value of CO_2_ permeance at high CO_2_ concentration reached in CO_2_/CH_4_ separation, may be correlated to the robustness of the 20 wt.% Zeolite A-PTMSP/P84 MMCHF membrane, attributed to the compatibility of the membrane materials components [30] without needing surface modification of the zeolites [48].

### 3.2. Model Validation

The agreement with the model used in this work is a function of the type of selective membrane material. For the commercial PDMSXA-10, it was acceptable, except at high CO_2_ concentration in the feed. There, the deviations can be attributed to the plasticizing effect by CO_2_ and the competing and synergistic interaction between the penetrants and the polymer matrix that is the base component of the selective layer of a composite membrane, causing a decrease in slow gas permeance at increasing CO_2_ concentration [18,49].

The calculated permeance of the MMCHF takes into account the consideration of the presence of water vapor in the feed, by introducing the Equation (4) obtained in a previous work [21], into the model.

The model prediction for this work CHF membranes is more accurate than in the case of the commercial PDMSXA-10 HF membrane, probably because it has been easier to consider the resistance in series of the different layers by Equation (3) and the effect of plasticization is not as significant as that of water vapor [49,50,51], given the low pressure difference of the separation experiments (75 psi in the feed side and 15 psi in the permeate side) [37].

**Figure 8:**
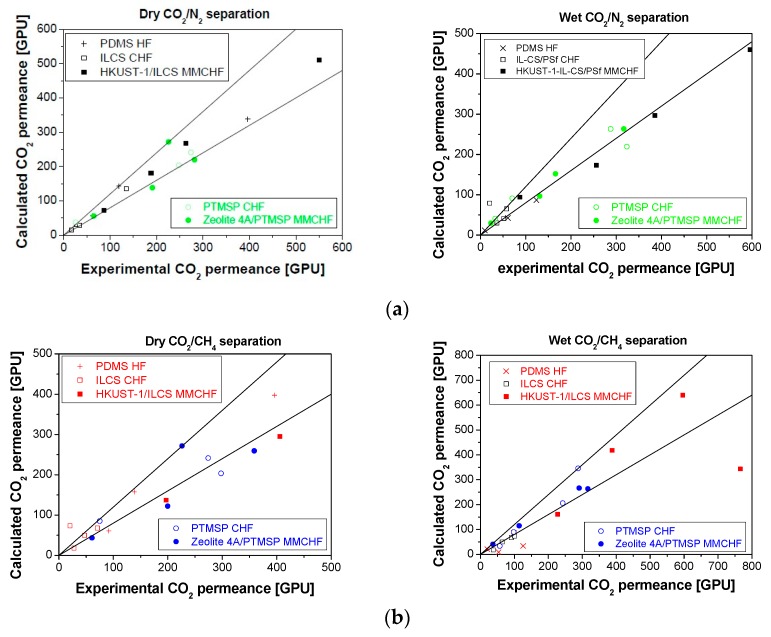
Parity plots for the CO_2_ permeance in dry (left) and humid conditions (right) obtained for the CO_2_/N_2_ (**a**) and CO_2_/CH_4_ (**b**) separation for all membranes tested in this work.

The experimental results were validated by the simple mathematical model incorporating Equations (3) and (4) to account for the change of geometry and the effect of humidity in the feed. The parity plots in Figure 8 show that this model predicts the CO_2_/N_2_ performance in dry conditions better, with an error below 20% (lines in Figure 8), while the CO_2_/CH_4_ behavior of the membranes is validated only in the presence of water vapor, with large deviations in dry conditions, as shown in the left-handed Figure 8a. The error in the CO_2_ permeate flux is also lower in wet than dry conditions, especially when the hydrophilicity of the selective CHF membrane can be tuned up [33].

The influence of water vapor in the feed stream on the permeation flux depends on the hydrophobic or hydrophilic character of the selective layer of the membrane, as this facilitates the affinity towards CO_2_ [45]. On the one hand, the permeance through the PDMSXA-10 and PTMSP membranes decreased in wet conditions, in response to the competitive sorption and transport through the hydrophobic membranes. On the other hand, the CO_2_ permeance through the IL-CS/PSf membrane increased in the presence of water vapor, with increasing CO_2_ concentration in the feed, which may be attributed to the increasing hydrophilic character of the selective layer [29].

The influence of water on CO_2_ solubility has been observed to alter the performance of other glassy polymer based MMMs [52]. Thus this model can still be enhanced in the future by considering recent advances taking into account the interfacial layer distance and distribution of the particles in the polymer matrix [53], as well as the water activity introduced in this work by Equation (4), in order to improve the accuracy of the model.

## 4. Conclusions

The experimental CO_2_ permeance and selectivity on the separation of CO_2_/N_2_ and CO_2_/CH_4_ mixtures, using several hollow fiber membranes, was measured in the absence and presence of water vapor in the feed. On the one hand, the permeance through the PDMSXA-10 HF and the PTMSP CHF membranes decreased in wet conditions in response to the competitive sorption and transport through the hydrophobic membranes. On the other hand, the CO_2_ permeance through the IL-CS/PSf membrane increased in the presence of water vapor, with increasing CO_2_ concentration in the feed. This was also observed for the HKUST-1-IL-CS/PSf and the zeolite A-PTMSP/P84 MMCHF membranes, which accounts for the possibility to tune up the CO_2_ separation by altering the hydrophilicity of the selective layer material in composite membranes. The experimental results have been validated by a simple mathematical model, adding the effect of the hollow fiber geometry and the influence of water activity in the gas feed, with a global error generally lower than 20%.

This work provides scope for the evaluation of novel CHF membranes in CO_2_ separation processes with non-ideal mixtures, to fill in the existing gap from laboratory to bench scale in the presence of impurities. Further characterization of the CO_2_ separation performance at higher pressures and in the presence of other impurities such as hydrocarbons in the absence and presence of water vapor conditions [51], both experimentally and theoretically, will allow for the evaluation of the potential of these membranes in biogas upgrading and other environmental applications.

## Figures and Tables

**Table 1:** Sequence of CO_2_ separation experiments conducted for each membrane.

Experiment ^1^	CO_2_ (wt.%)	N_2_ (wt.%)	CH_4_ (wt.%)	RH (%)
1	0	100	0	0
2	0	0	100	0
3	25	75	0	0
4	25	0	75	0
5	50	50	0	0
6	50	0	50	0
7	75	25	0	0
8	75	0	25	0
9	100	0	0	0
10	0	100	0	50
11	0	0	100	50
12	25	75	0	50
13	25	0	75	50
14	50	50	0	50
15	50	0	50	50
16	75	25	0	50
17	75	0	25	50
18	100	0	0	50

^1^ Noteworthy were the order of gas concentrations (N_2_, CH_4_, CO_2_) and dry and wet streams in order to assure reproducibility of the membrane materials between experiments, as reported elsewhere [18].

## Abbreviations

*a*	water activity, in Equation (4)
A	Effective membrane area, cm^2^
β_i_	Intrinsic gas pair selectivity
CHF	Composite hollow fiber
CS	Chitosan biopolymer
Δ*p_i_*	partial pressure difference for the gas component *i*
D	Diffusivity, cm^2^ s^−1^, in Equation (4)
FFV	Fraction of free volume
GPU	Units of permeance, 10^−6^ cm^3^(STP)·cm^−2^ s^−1^ cmHg^−1^
IL	Ionic liquid; in this work, 1-ethyl-3-methylimidazolium acetate
MMCHF	Mixed matrix composite hollow fiber
P84	BTDA-TDI/MDI, 3,3′4,4′-benzophenone tetracarboxylic dianhydride and 80% methylphenylene-diamine + 20% methylene diamine copolyimide
PSf	Polysulfone polymer
PTMSP	Poly[1-(trimethylsilyl)-1-propyne]
Q	Gas flow rate, cm^3^ s^−1^
t	selective layer thickness
RH	Relative Humidity, %
S	Solubility, cm^3^(STP) cm^−3^·g, in Equation (4)
